# Respiratory syncytial virus infections – recent developments providing promising new tools for disease prevention

**DOI:** 10.2807/1560-7917.ES.2023.28.49.2300686

**Published:** 2023-12-07

**Authors:** Eeva K Broberg, Hanna Nohynek

**Affiliations:** 1 European Centre for Disease Prevention and Control (ECDC), Stockholm, Sweden; 2 Finnish institute for health and welfare, Helsinki, Finland

**Keywords:** Respiratory syncytial virus, monoclonal antibody, prevention, infection, whole genome sequencing

After 60 years of development, respiratory syncytial virus (RSV) vaccines intended for large scale use both in elderly people and pregnant women have recently obtained regulatory approval in the European Union (EU) [1]. Aside from this important breakthrough, a further milestone in the prevention of RSV infections was reached in October 2022, when a long-acting monoclonal antibody nirsevimab was licensed in the EU for use in infants [2].

Respiratory syncytial virus infections are a major cause of hospitalisation in children worldwide, resulting in the death of an estimated 101,400 children below 5 years of age annually [3]. In most European countries, RSV is not (yet) notifiable and therefore, accurate figures for surveillance and burden of disease indicators (and comparisons with past seasons) cannot be easily drawn from the available data. However, a recent study by Wildenbeest et al. following nearly 1,000 newborns over the first year of life in a European context, estimated an incidence of RSV hospitalisations in normal term babies at 1.8% (95% CI: 1.6–2.1) and 5.5% of them to be admitted to intensive care units (ICUs) [4]. A substantial number of hospitalisations in children younger than 3 years of age in Europe are due to RSV, with the majority being in the age group 0–2-months-old and above, with more than 40 per 1,000 children in this age group being hospitalised due to RSV annually [5]. Recurrent wheeze or asthma hospitalisations have been associated as long-term sequelae of RSV infection in children with a cumulative incidence rate of 16.6 per 1,000 person-years after RSV hospitalisation of infants younger than 6 months of age [6]. For European adult populations, RSV is an important cause of lower respiratory tract infections and 92% of hospitalisations due to RSV occur in adults aged 65 years and older [7]. Among people aged 85 years and above, the annual hospitalisation rate due to RSV is three per 1,000 persons in that age group [7].

Due to the high burden of disease caused by RSV, the differential diagnosis of RSV from other respiratory viruses that cause similar acute respiratory infections is crucial. For this purpose, using multiplex PCRs detecting multiple respiratory viruses simultaneously is key [8]. Moreover, to understand the annual circulation patterns and genomic evolution of RSV, sequencing data need to be collected and reported to publicly accessible databases. As for other respiratory viruses and most infectious diseases, under-ascertainment will remain an issue for RSV surveillance in general as patients may present late for testing after the infection or not even be tested if they present with mild symptoms.

In this issue of *Eurosurveillance*, Iglesias-Caballero et al. [9] describe two PCR-based sequencing systems for RSV, one for generic whole-genome sequencing of RSV subtype A and B and one that targets the RSV major antigens G and F. Especially G gene is used for genotyping purposes due to its higher variability, while the F gene represents a more conservative gene area and is therefore used for vaccine development [10]. The authors demonstrated that both methods can be used for clade assignment of RSV A and B subtypes with 100% agreement. However, it has been shown earlier that using only the G gene for genotyping, can lead to missing some genotypes where there is variability in other RSV genes [10]. Several other methods have already been developed for RSV sequencing. The advantage of the presented approach is that complete genomes can be obtained directly from clinical specimens with even low viral loads and without requiring prior subtyping and quantification steps. Therefore, this method is less labour-intensive and less costly, and it could increase laboratory capacities and probably also shorten throughput times. The study demonstrated that it can be used in retrospective molecular epidemiological studies of RSV and even with the simplified approach to sequence only the G and F gene genome, molecular evolution of RSV could be assessed.

Another article in this issue, by Martinón-Torres et al. [11], reviews the early lessons from Galicia, Spain, on the implementation of universal RSV prophylaxis in infants with long-acting monoclonal antibodies. Spain and France are the European countries which already introduced nirsevimab in their national immunisation programmes [12]. Nirsevimab is the first long-acting anti-RSV monoclonal antibody which received authorisation in the EU recently [2]. Several other anti-RSV monoclonal products as well as vaccines are currently being developed [13]. The early uptake of nirsevimab, ranging between 81–98% in Galicia thus far, has exceeded expectations. This success is the result of carefully planned target groups and rationally designed immunisation logistics. The memory of the very intense recent RSV epidemic in Europe during the autumn of 2022 along with awareness raising communications likely helped motivate parents to have their children immunised [14]. The authors justify the universal introduction of nirsevimab into the childhood vaccination programme by careful examination of the burden of disease in the region and the potential impact and efficiency of the intervention [15] while there was no reference to a formal cost-effectiveness analysis or health technology assessment as a requirement before introduction. This is interesting as in many EU countries in non-pandemic situations, decision-makers usually require at least reasonable cost-effectiveness of the preventive intervention before agreeing upon its public funding. In the European context, such health economic models have been constructed and used in analytic work [16,17]. There have been concerns that with the present prices of the product, a universal introduction of nirsevimab may be too expensive to justify public spending.

Thus, the value of the work of Martinón-Torres et al. lies not only in the clear and meticulous description of how implementation challenges were approached, i.e. by distinguishing infants belonging to the seasonal and catch-up or high-risk groups, which provides an excellent example to other European countries, but also in the fact that Galicia will be able to answer the burning question on whether universal introduction of nirsevimab in the real-world setting is a cost-effective intervention on the short as well as the long run. If preventing RSV infection in early life can reduce asthma later in life, the impact of the intervention will be manifold.

Still, another timely question is whether the monoclonal approach, as designed by the Galicia public health authorities, is superior or equal to the alternative approach of vaccinating pregnant women. To provide answers, further modelling work is needed before countries are willing to take major steps in their decision making.

The high healthcare burden caused by RSV infections in young children could be reduced by vaccination of pregnant women or immunisation of healthy term-born infants with a monoclonal antibody during their first winter season as it has been shown that vaccination of pregnant women in the latter part of pregnancy or alternatively giving nirsevimab to the infant before the RSV season significantly reduced the risk of medically-attended and hospitalisation for RSV-associated lower respiratory tract infection [18,19].

COVID-19 pandemic has highlighted the need to invest in respiratory disease surveillance on a year-round basis with strong syndromic and genomic monitoring components to allow continuous checking of severe disease indicators and variant evolution in relation to the vaccine strains. The European Centre for Disease Prevention and Control and the World Health Organization Regional Office for Europe have agreed on an integrated respiratory surveillance [8], which also includes RSV, and the weekly results are published in the European Respiratory Virus Surveillance Summary [20]. Severe acute respiratory infection (SARI) sentinel surveillance serves as one possible syndromic surveillance system for capturing RSV cases in hospitals. Age-specific ICU and mortality monitoring are important parts of RSV surveillance to capture the whole spectrum of disease burden in Europe in addition to the primary care sentinel surveillance and secondary care laboratory notifications.

The RSV vaccines available today will not be the only solution of prevention strategies. The RSV vaccine development pipeline revolutionised by vaccine technology breakthroughs to control COVID-19 is promising [13]. If all goes well, we will not only see RSV vaccines in solo, but possibly combination of vaccines with RSV and/or influenza and/or COVID-19 in one shot. Despite the possible bright future, countries are struggling with resources to finance such introductions. Therefore, cost-effectiveness and impact analyses are needed to help national decision-makers.

Implementation of vaccine and immunisation programmes for RSV call for enhanced RSV surveillance at sentinel GP and especially at hospital level. Implementation of genomic surveillance for RSV will also require considerable efforts in the national surveillance systems, however, these efforts are crucial for monitoring vaccine escape variants, development of possible resistance to antibody therapies and selecting future RSV vaccine antigens.
